# A novel polysaccharide in the envelope of *S. aureus* influences the septal secretion of preproteins with a YSIRK/GXXS motif

**DOI:** 10.1128/jb.00478-24

**Published:** 2025-01-28

**Authors:** Amany M. Ibrahim, Dominique Missiakas

**Affiliations:** 1 Department of Microbiology, Howard Taylor Ricketts Laboratory, The University of Chicago 456539 , Chicago, Illinois, USA; The Ohio State University, Columbus, Ohio, USA

**Keywords:** *Staphylococcus aureus*, polysaccharide, teichoic and teichuronic acid, cross-wall, septal membrane, protein secretion, YSIRK/GXXS motif

## Abstract

Bacteria transport proteins across the plasma membrane to assemble their envelope, acquire nutrients, and establish appropriate interactions with their environment. The majority of these proteins are synthesized as precursors with a cleavable N-terminal signal sequence for recognition by the Sec machinery. In *Staphylococcus aureus*, a small subset of secreted precursors carries a YSIRK/GXXS motif. This motif provides a pre-translocation function by promoting the targeting of precursors to septal membranes, but the *trans-*acting factors that regulate such spatial distribution are not known. Here, we used immunofluorescence-microscopy to compare the spatial trafficking of Staphylococcal protein A (SpA), an abundant YSIRK/GXXS bearing precursor, between mutants of an arranged transposon library. This genetic search identified a cluster of five genes predicted to encode enzymes responsible for the synthesis of a novel surface polymer referred to as Staphylococcal surface carbohydrate, Ssc. Mutants in the *ssc* gene cluster no longer restrict the secretion of SpA into the cross-walls of *S. aureus. ssc* mutants replicate like wild-type bacteria unless grown in phosphate-limited conditions, and do not contribute to virulence when examined in a mouse model of bloodstream infection. Together, our observations suggest that *S. aureus* may encode a minor, phosphate-free carbohydrate, and propose a possible assembly pathway for this polymer.

**IMPORTANCE:**

Gram-positive bacteria assemble peptidoglycan-linked polymers known as wall teichoic acids (WTA). Both *Staphylococcus aureus* and *Bacillus subtilis* elaborate WTAs made of poly-glycerol or poly-ribitol phosphates. WTAs contribute to cell shape maintenance, cation homeostasis, and resistance to antimicrobial compounds. Yet, *B. subtilis* replaces its phosphate-rich polymer with minor teichuronic acids whose functions remain elusive. *S. aureus* also encodes a minor wall polymer that may be required for growth under phosphate-limited condition. Here, we find that this polymer could help define the composition of the septal compartment, the site of cell division also used to recruit preproteins with a YSIRK/GXXS motif. Thus, the envelope of *S. aureus* may be more complex than previously thought with minor wall polymers contributing some discrete functions.

## INTRODUCTION

*Staphylococcus aureus* is a typical gram-positive organism endowed with a plasma membrane and surrounded by a thick layer of peptidoglycan. *S. aureus* bacteria are spherical and divide at the mid-cell where the new envelope is assembled via invagination, a process that is microscopically identified as a septum or cross-wall. Septum-splitting by dedicated peptidoglycan hydrolases yields two equally sized daughter cells (1–4). As with many other organisms, septum placement is governed by FtsZ, a conserved protein that oligomerizes into the so-called Z-ring (5, 6). Z-ring formation marks the recruitment of other “divisome” proteins that drive the completion of the bacterial cell cycle. Divisome components vary between species to accommodate for differences in shape and envelope composition (7–9). In *S. aureus*, envelope complexity arises with the incorporation of lipoteichoic acid (LTA) in the outer leaflet of the plasma membrane and with peptidoglycan substitutions including wall teichoic acid (WTA), capsular polysaccharide, and surface proteins (10–18). At least three of these molecules, LTA, WTA, and surface proteins with a conserved YSIRK/GXXG motif, have been proposed to be assembled at, or trafficked to, septal locations (19–24). We reported earlier that the genetic disruption of LTA assembly results in the loss of septal secretion (22), and proposed a mechanism that may account for the restricted assembly of LTA and YSIRK/GXXG protein secretion into septal compartments (24).

Signal (leader) peptides identify proteins destined for secretion or export by allowing selective interactions with cognate translocases in the cytoplasmic membrane (25–27). Biochemical and genetic studies have shown that bacterial secretion (Sec) signal peptides engage with the SecA translocase to facilitate the threading of unfolded proteins across the narrow membrane translocon formed by the SecYEG complex (28–32). In *S. aureus* and other gram-positive bacteria, some proteins carry an extended signal peptide with the conserved YSIRK/GXXG sequence element that is necessary and sufficient to direct secreted precursors to septal membranes (19, 33, 34). YSIRK/GXXG-containing signal peptides are still recognized by SecA (22, 35), but the *trans*-acting factors responsible for their septal targeting remain unknown. Many YSIRK/GXXG precursors are surface proteins that contain a C-terminal cell wall sorting sequence with the conserved LPXTG motif. This motif is cleaved by the enzyme Sortase A on the *trans* side of the membrane between the threonine and glycine residues (36–38). Sortase A subsequently links this cleaved product to the pentaglycine cross-bridge of newly synthesized peptidoglycan subunits immobilizing proteins on the cell surface (36, 39, 40). This tethering affords visualization by microscopy (19). When cells of *S. aureus* are treated with trypsin, all surface-exposed proteins are removed from the bacterial envelope, and the trafficking of newly synthesized proteins can then be monitored using immunofluorescence microscopy (19). Using this assay, it was found that proteins endowed with the canonical secretion signal, such as SasA, SasF, or SasK, are deposited at the cell poles and appear as foci on the cell surface, while those harboring the YSIRK/GXXG motif, such as SpA, ClfA, ClfB, FnbpB, SdrC, and SdrD, are trafficked to the septal compartment (19). Since *S. aureus* divides symmetrically, upon completion of the cell cycle, YSIRK/GXXG surface proteins appear evenly distributed over half of the surface of newly separated daughter cells (19, 22, 24).

In this study, we used confocal fluorescence microscopy to screen an arranged transposon library for mutants that fail to restrict SpA secretion into the cross-walls of dividing *S. aureus*. By systematically walking along the chromosome of *S. aureus*, we identified a cluster of five genes predicted to synthesize an uncharacterized polysaccharide. To validate our findings, we generated three mutants by allelic replacement and confirmed that mutational lesions in this gene cluster result in the loss of septal secretion of SpA. During the course of our investigations, Lei et al. reported the characterization of a new surface polymer that they named Staphylococcal surface carbohydrate, Ssc (41). This polymer is the same as the one uncovered in our study, and thus, we have adopted the Lei et al. nomenclature to describe the *ssc* gene cluster. Our data reveal that in rich media, but not when phosphate is limiting, Ssc is dispensable for growth. Yet, *ssc* mutants retain full bacterial virulence in a bloodstream mouse model of infection. *ssc* mutants do not affect the assembly of LTA. Thus, the inability of *ssc* mutant to restrict septal secretion of proteins cannot be explained by an indirect effect on LTA. While we cannot readily explain how the presence of the Ssc polymer may help distinguish septal membranes, we propose a model for the assembly of this polymer in *S. aureus* based on bioinformatic predictions.

## RESULTS

### A screen for mutants with defect in septal trafficking of Staphylococcal protein A (SpA) identifies a *tua*-like gene cluster

In an attempt to find cellular factors that contribute to the trafficking of YSIRK/GXXG proteins, we monitored the secretion of the abundant cell surface protein, SpA, using fluorescence microscopy and screened an arranged collection of transposon mutants derived from the Phoenix library in strain Newman (42, 43). This approach identified a cluster of five genes, *nwmn_0072* to *nwmn_0076*, within the first 100 genes of the chromosome of strain Newman (44). The bioinformatic analysis of this cluster suggested that the five genes may encode enzymes for the synthesis of an envelope polysaccharide that was recently reported as the staphylococcal surface carbohydrate, *ssc* gene cluster, by Lei and colleagues (41). The rationale for this naming was based on the finding that expression of the *ssc* genes in *S. aureus* affected phage adsorption and susceptibility, while expression of the *ssc* genes in *Escherichia coli* resulted in the synthesis of polymers containing N-acetylgalactosamine (41). Thus, we have retained the Ssc nomenclature. *S. aureus sscA* is predicted to encode an NAD-dependent epimerase reminiscent of GalE, an enzyme that reversibly converts UDP-glucose (UDP-Glu) to UDP-galactose (UDP-Gal). *sscB* and *sscC* are predicted to encode a membrane-bound and a cytoplasmic glycosyltransferase, respectively, while SscD and SscE appear to be highly hydrophobic with 12 and 13 predicted membrane segments and with proposed functions as polymerase and transporter (Table 1). When the BLASTP search was performed against *Bacillus subtilis,* SscC, SscD, and SssE could be matched with TuaC, TuaE, and TuaB, respectively (Table 1). In *B. subtilis* strain 168, the *tua* gene cluster is arranged as an operon of eight genes, *tuaABCDEFGH* (45), and synthesizes a polymer known as teichuronic acid composed of disaccharide repeats of glucuronyl-N-acetylgalactosamine (46). A closer examination suggested that *sscB* may encode a UDP-phosphate N-acetylgalactosaminyl-1-phosphate transferase reminiscent of TuaA, the committing enzyme of teichuronic acid synthesis. However, in some strains of *B. subtilis,* including 168, the *tuaA* gene is a pseudogene, and a match with *sscB* was not readily observed. Together, this bioinformatic analysis suggests that *S. aureus* may produce a wall polymer distinct of teichoic acid but reminiscent of teichuronic acid found in other Firmicutes.

**TABLE 1:** Predicted function and cellular location of *ssc* gene products

Locus tag in Newman	Product	Length (aa)^*a*^	Predicted location^*b*^	Predicted function^*c*^	Match^*d*^	Proposed function^*e*^
NWMN_0072	SscA	319	Cytoplasm	Rossmann-fold NAD(P)(+)-binding proteins NAD-dependent epimerase/ dehydratase family protein	GalE E value: 6e − 44 Identity: 31%	UDP-glucose 4-epimerase: interconverts UDP-Glc and UDP-Gal
NWMN_0073	SscB	230	Membrane 1 TM	WcaJ-like Sugar transferase COG2148	TuaA E value: 6e − 76 Identity: 56%	UDP-phosphate N-acetylgalactosaminyl-1-phosphate transferase: initiator enzyme
NWMN_0074	SscC	388	Cytoplasm	Glycosyl transferase, group 1 family protein GT4_CapM-like	TuaC E value: 9e − 11 Identity:32%	Glycosyltransferase: uses UDP-sugar as a substrate
NWMN_0075	SscD	412	Membrane 12 TM	RfaL O-antigen ligase COG3307	TuaE-like	Polymerase
NWMN_0076	SscE	476	Membrane 13 TM	Polysaccharide extrusion protein (MATE_like)	TuaB E value: 2e − 05 Identity:23%	Transport

^*a*^ Length of protein in amino acids (aa).

^*b*^ Topology and subcellular location predictions performed with PSORTb version 3.0.3 (https://www.psort.org/psortb/) and DeepTMHMM - 1.0 (https://services.healthtech.dtu.dk/services/DeepTMHMM-1.0/)

^*c*^ Function according to domain annotation in the NCBI Databank.

^*d*^ Match according to a BLASTP search against *Bacillus subtilis* genomes.

^*e*^ Proposed functions.

### Expression of *ssc* genes and phenotypic characterization of *ssc* in-frame deletion mutants

To ascertain that the *ssc* genes are expressed in the experimental growth conditions used in our screen, we conducted a semi-quantitative PCR with primers specific to each gene. Messenger RNA was extracted from cells from mid-log phase cultures grown at 37°C in tryptic soy broth to generate a cDNA library via reverse transcription (Fig. S1A). As a negative control, reverse transcriptase was omitted from some reactions, while primers for *spa* gene expression were included as a positive control. This approach confirmed the expression of the *ssc* gene cluster (Fig. S1A).

**Fig 1:**
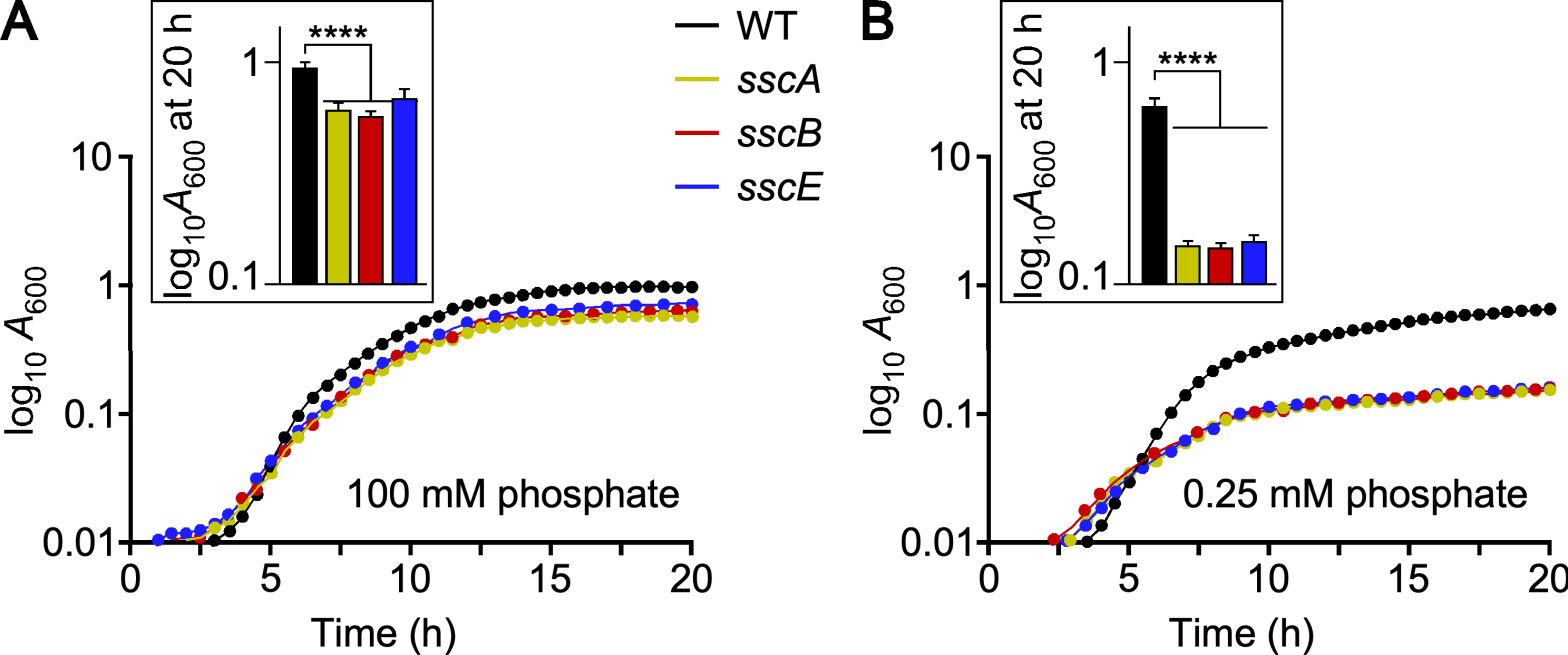
Assessing the growth of *ssc* mutants under phosphate-limited conditions. Overnight cultures grown in modified M63 medium containing 100 mM K_2_HPO_4_ were diluted (1:100) in the same medium (A; 100 mM K_2_HPO_4_) or medium containing 0.25 mM K_2_HPO_4_ (**B**). Growth was monitored as changes in absorbance at 600 nm (*A*_600_), recorded in 30 min intervals for 20 h and in triplicate. A representative experiment is shown. Insets show *A*_600_ recordings at 20 h.

Since our screen was conducted using transposon mutants that could exert polar effects on downstream genes, we used allelic replacement to delete the coding sequences of *sscA*, *sscB,* and *sscE* in two wild-type strains, Newman (NWMN) and RN4220. In the case of *sscA*, the spectinomycin resistance gene was inserted in place of the coding sequence. No growth defect was noted upon deletion or replacement of the three genes (Fig. S1B and C). When bacteria were grown in modified M63 medium, a defined rich medium that supports slower replication compared to tryptic soy broth (47), all three *ssc* mutants, *sscA*, *sscB,* and *sscE*, grew slightly more slowly than wild type in the presence of 100 mM phosphate (Fig. 1A). The growth defect was further exacerbated when cultures were grown in phosphate-limited conditions, modified M63 medium supplemented with 0.25 mM phosphate (Fig. 1B).

**Fig 2:**
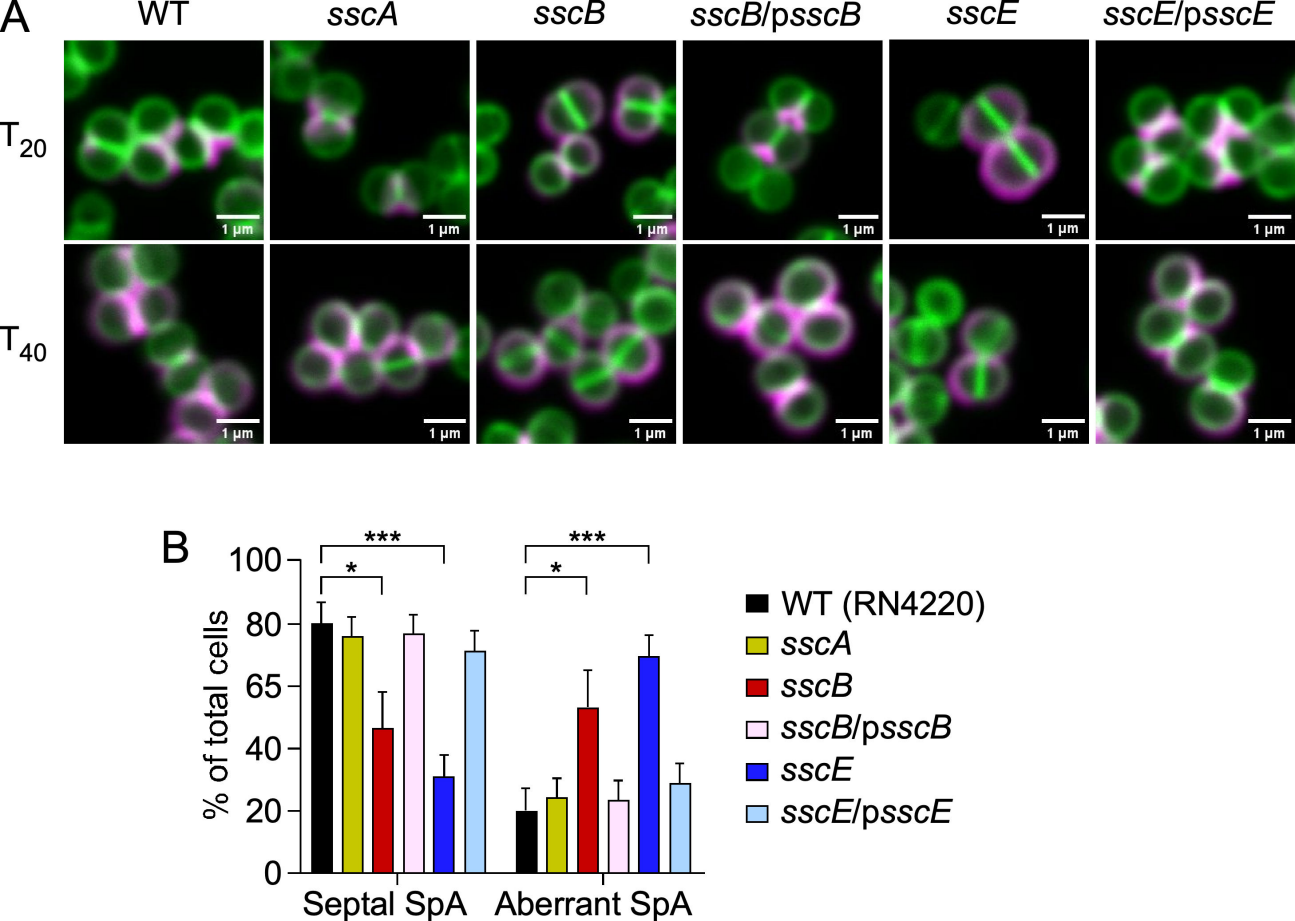
Trafficking of SpA is no longer restricted to the cross-walls of *S. aureus* in the *sscB* and sscE mutants. (**A**) Cells were treated with trypsin to remove surface proteins, and the distribution of SpA in the cell wall envelope was examined after addition of trypsin inhibitor for 20 and 40 min (*T*_20_/*T*_40_). Fluorescence microscopy images were obtained using BODIPY FL-vancomycin (green) to visualize the cell wall and SpA-specific antibodies followed by secondary goat anti-human conjugated to Alexa Fluor 647 (magenta). Two-dimensional two-color images were acquired using a Stellaris 5 confocal microscope. Scale bars = 1 µm. (**B**) Immunofluorescent SpA signal was calculated from at least three independent experiments as the percentage of cells with septal or aberrant SpA deposition with respect to the total number of cells counted. Data were analyzed using two-way analysis of variance with Dunnett’s multiple comparison test (*P = 0.0157; ***P = 0.0004).

Next, septal anchoring of SpA was assessed using fluorescence microscopy to compare the three *ssc* mutants with wild-type RN4220. First, trypsin was used to remove surface proteins from wild-type and mutant bacteria, then trypsin inhibitor was added for 20 or 40 min (*T*_20_/*T*_40_) to allow for cell wall deposition of newly synthesized proteins following one or two cell division cycles (19, 22, 24). To visualize SpA trafficking, cells were labeled with SpA-specific monoclonal antibody and Alexa Fluor (AF) 647-conjugated secondary IgG (magenta) followed by BODIPY FL-vancomycin (green), which binds to the terminal D-alanyl-D-alanine moieties of peptidoglycan. As expected, SpA immune signals were observed in the splitting cross-walls of wild-type cells at *T*_20_ with a more extended surface distribution at *T*_40_ (Fig. 2A). Disrupting *sscA* did not affect SpA trafficking, but in the absence of *sscB* or *sscE*, SpA secretion was no longer restricted to the cross-walls at *T*_20_ (Fig. 2A). This aberrant trafficking was, however, restored to the wild-type pattern upon introduction of complementing plasmids encoding *sscB* or *sscE* (Fig. 2A and B).

**Fig 3:**
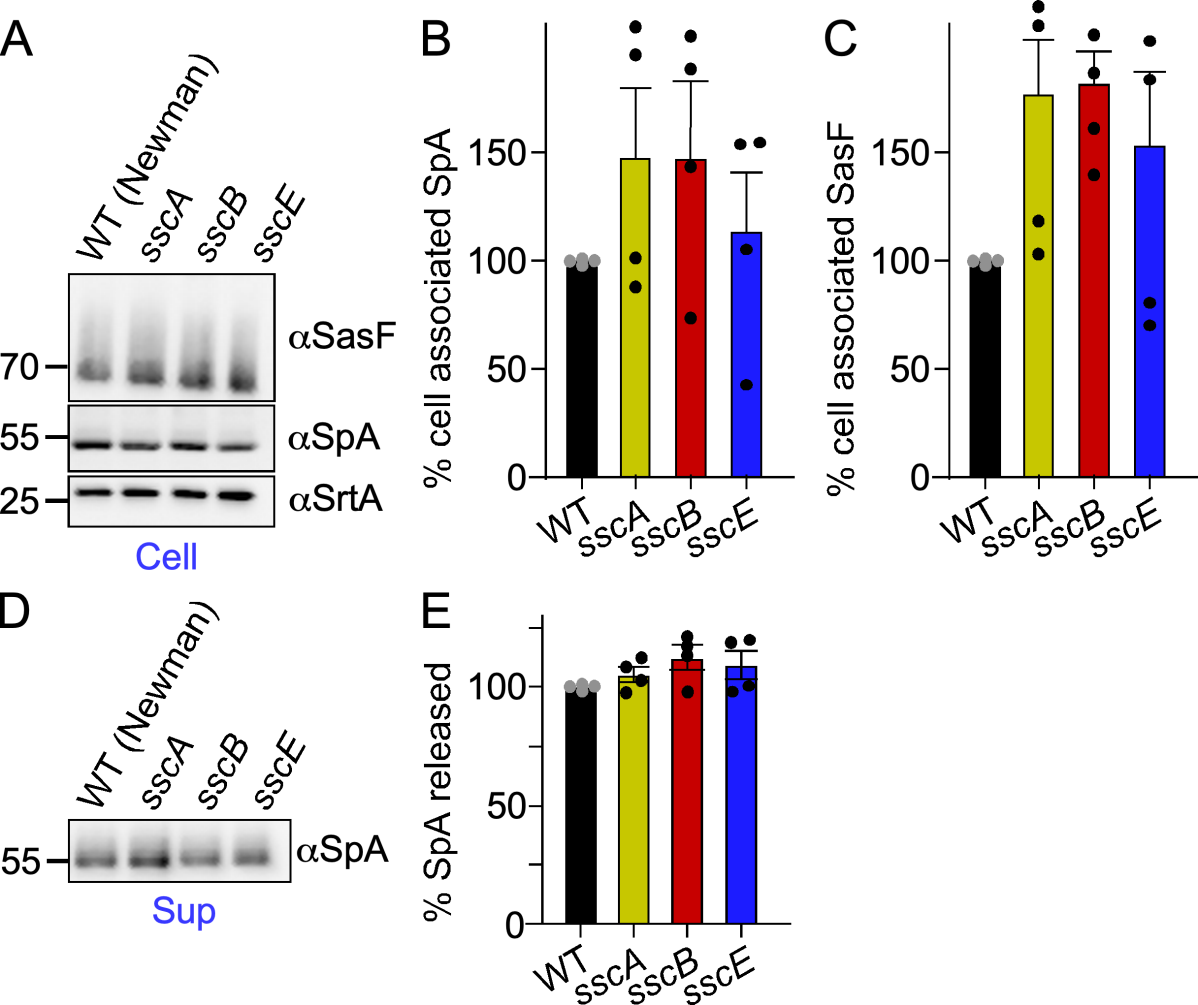
Genetic disruption of the Ssc biosynthesis pathway does not affect surface anchoring of proteins or their extracellular release. (**A**) Cell-associated SpA and SasF were identified by immunoblot in cell sediments (Cell) following treatment with lysostaphin. Following transfer to blots, immune detection was performed using antibodies against SpA (αSpA), SasF (αSasF), and sortase A (αSrtA) as a loading control. Densitometry quantification of immunoreactive signals of cell-associated SpA (**B**) and SasF (**C**). (**D, E**) Bacterial cultures shown in (**A**) were spun to quantify the amount of SpA released in the extracellular milieu (Sup, culture supernatant). The blot was scanned to quantify immune signals of released SpA (**E**). All immunoblot experiments were performed four times independently. No statistical differences were found.

To rule out the possibility that *ssc* genes may grossly impact the secretion and sorting of proteins, cultures of wild-type (Newman) and *ssc* mutant bacteria were sedimented to separate cells from medium (culture supernatant). Cells were treated with lysostaphin to solubilize peptidoglycan-anchored proteins. Samples were subjected to immunoblotting using antibodies specific for SpA and SasF, another cell wall-anchored protein that lacks a YSIRK/GXXG motif (Fig. 3A through C). The presence of SpA was also examined in culture supernatants as it is released from the cell by the action of the LytN and LytM hydrolases (48). Immunoblotting did not reveal any changes in the amounts of SpA and SasF associated with the cells (Fig. 3A through C), nor in the amount of SpA released in the extracellular fraction (Fig. 3D and E). Together, these results suggest that the product of the *ssc* gene cluster is important for restricting septal secretion of SpA, but this novel polymer does not affect protein anchoring in the envelope.

### *ssc* mutant bacteria do not display any defect in LtaS processing or LTA assembly

SscA is predicted to encode a UDP-Glc 4-epimerase implying that the Ssc pathway could share the pool of UDP-Glc with the LTA assembly pathway. Two molecules of UDP-Glc are used by the enzyme YpfP to generate diglucosyldiacylglycerol, Glc_2_-DAG (14, 49). Once flipped to the *trans* side of the plasma membrane by LtaA, Glc_2_-DAG serves as the starting unit for the stepwise addition of 15–50 *sn*-glycerol-1-phosphate (Gro-P) units by LtaS (15). In the absence of Glc_2_-DAG, when *ypfP* is not expressed, LtaS assembles longer (Gro-P) chains on diacylglycerol (DAG) (14, 50). In the absence of Glc_2_-DAG, the processing of LtaS to extracellular LtaS (eLtaS) by signal peptidase is also impaired. LtaS remains membrane-tethered throughout the cell cycle and continues to elongate (Gro-P) chains which also correlates with the loss of YSIRK/GXXG protein trafficking at the cross-walls (24).

**Fig 4:**
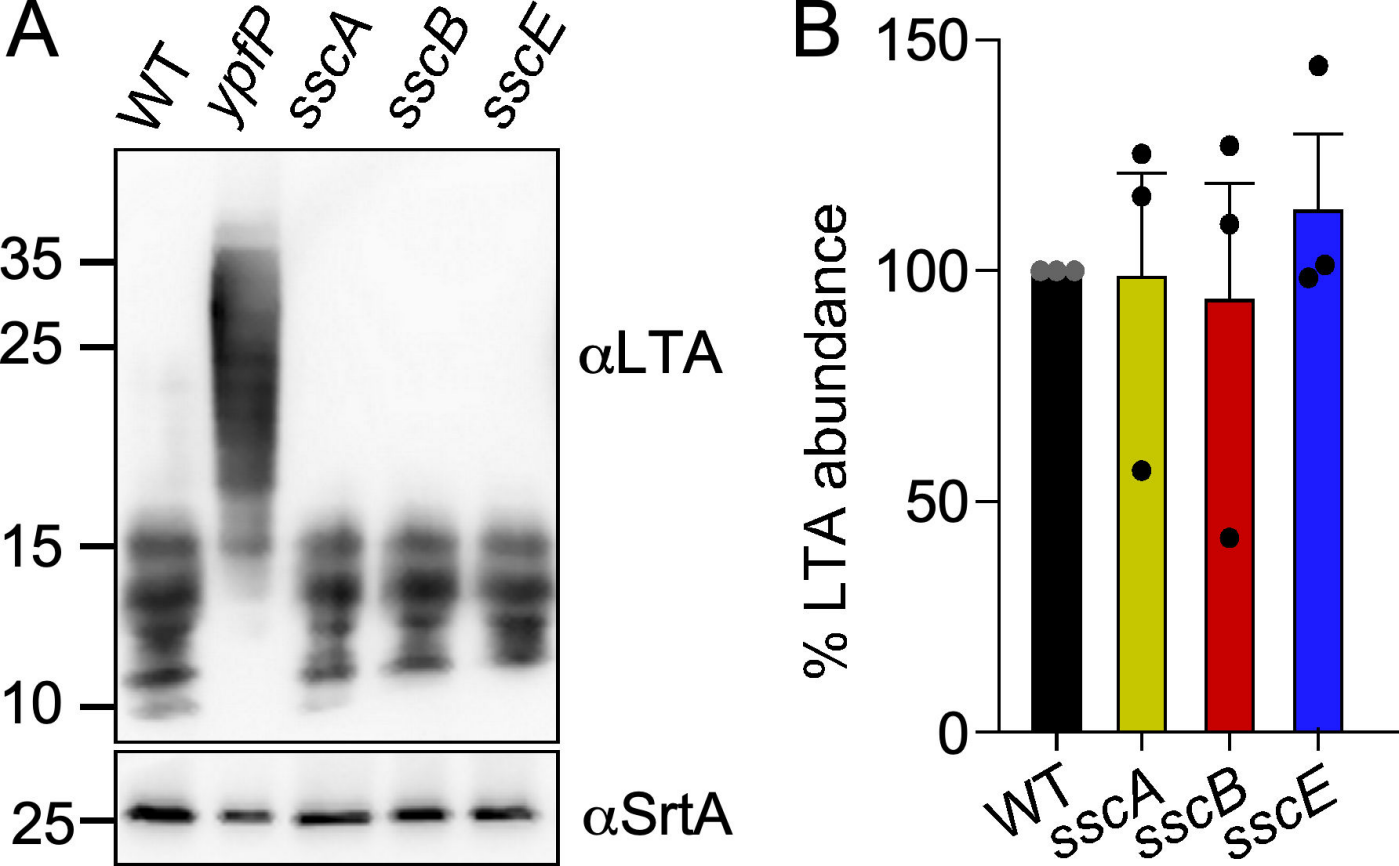
LTA chain length and abundance are not changed in *ssc* mutant**s**. LTA production was examined by immunoblotting samples from washed cells (**A**) Blots were analyzed with antibodies against LTA (αLTA) and sortase A (αSrtA) as a loading control. An *ypfP* mutant was included to document an example of aberrant migration of LTA. Experiments were performed at least three times. The sizes of the MW markers are shown to the left of each blot. (**B**) Quantification of LTA signals shown in (**A**) suggested no significant changes in the abundance of the polymer.

If both SscA and YpfP use the UDP-Glc pool, we wondered whether gene deletions in the *ssc* cluster that impact YSIRK/GXXG protein trafficking do so indirectly by altering the length of LTA or the processing of eLtaS. Immunoblotting of bacterial extracts was used to examine these possibilities (Fig. 4). LTA was extracted from stationary phase cultures and subjected to SDS-PAGE separation followed by immunoblotting with LTA-specific monoclonal antibodies. Unlike with the *ypfP* mutant, the chain length of LTA molecules was not altered in the *sscA*, *sscB*, or *sscE* mutants and the *ssc* mutants produced similar amounts of LTA as compared to the wild-type strain Newman (Fig. 4A and B). Similarly, the presence of LtaS and eLtaS was detected by immunoblotting bacterial extracts prepared from lysostaphin-lysed cultures. We did not observe any impairment in the processing of the LtaS enzyme in the absence of *sscA*, *sscB*, or *sscE* (Fig. S2). We conclude that the inability to restrict SpA trafficking to the cross-walls of dividing cells in the *sscB* and *sscE* mutants is not caused by the aberrant assembly of LTA that may have been caused by a shared pool of precursor molecules with the Ssc polymer.

### *S. aureus ssc* mutants did not display any major changes in susceptibility toward antibiotics

The presence of WTA and LTA has been reported to modulate the susceptibility of staphylococcal cells to antibiotics (51, 52). Thus, we asked if the purported Ssc polymer could also provide a layer of protection against three cell wall-targeting antibiotics, vancomycin, amoxicillin, bacitracin, and the protein synthesis inhibitor, linezolid, used to treat infections caused by antibiotic-resistant gram-positive bacteria such as MRSA (methicillin-resistant *S. aureus*) and VRE (vancomycin-resistant enterococci). We noticed a minor increase in resistance toward vancomycin for all three mutants, *sscA, sscB, sscE*, as compared to Newman (Table 2). This result corroborates with the slightly reduced (but not statistically different) intensities of the BODIPY-vancomycin channel observed in images shown in Fig. 2A and quantified in Fig. S3. All three *ssc* mutants were slightly more sensitive to amoxicillin, while mutants lacking SscB and SscE (but not SscA) displayed reduced minimum inhibitory concentrations (MICs) against daptomycin. No changes were observed with bacitracin. Lastly, the *sscE* mutant appeared to be slightly more resistant toward linezolid as compared to the wild-type and *sscA* or *sscB* mutant strains (Table 2). We conclude that loss of *ssc* gene expression results in subtle changes in the envelope that do not impact the susceptibility or resistance to antimicrobial compounds. Whether this is a direct effect due to the absence of the polymer or other compensatory mechanism remains to be determined.

**TABLE 2:** Antibiotic susceptibility of various *ssc* mutants

	Minimum inhibitory concentration (MIC^*a*^, μg/mL) for				
**Strain**	Vancomycin	Amoxicillin	Bacitracin	Linezolid	Daptomycin
Newman	1	0.125	128	2	1
*sscA*	2	0.094	128	2	1
*sscB*	2	0.094	128	2	0.75
*sscE*	1.5	0.094	128	3	0.75

^*a*^ The MIC of each antibiotic is the mean determined from at least three experiments performed in duplicate.

### *S. aureus ssc* mutants do not display reduced virulence in a murine model of bloodstream infection

**Fig 5:**
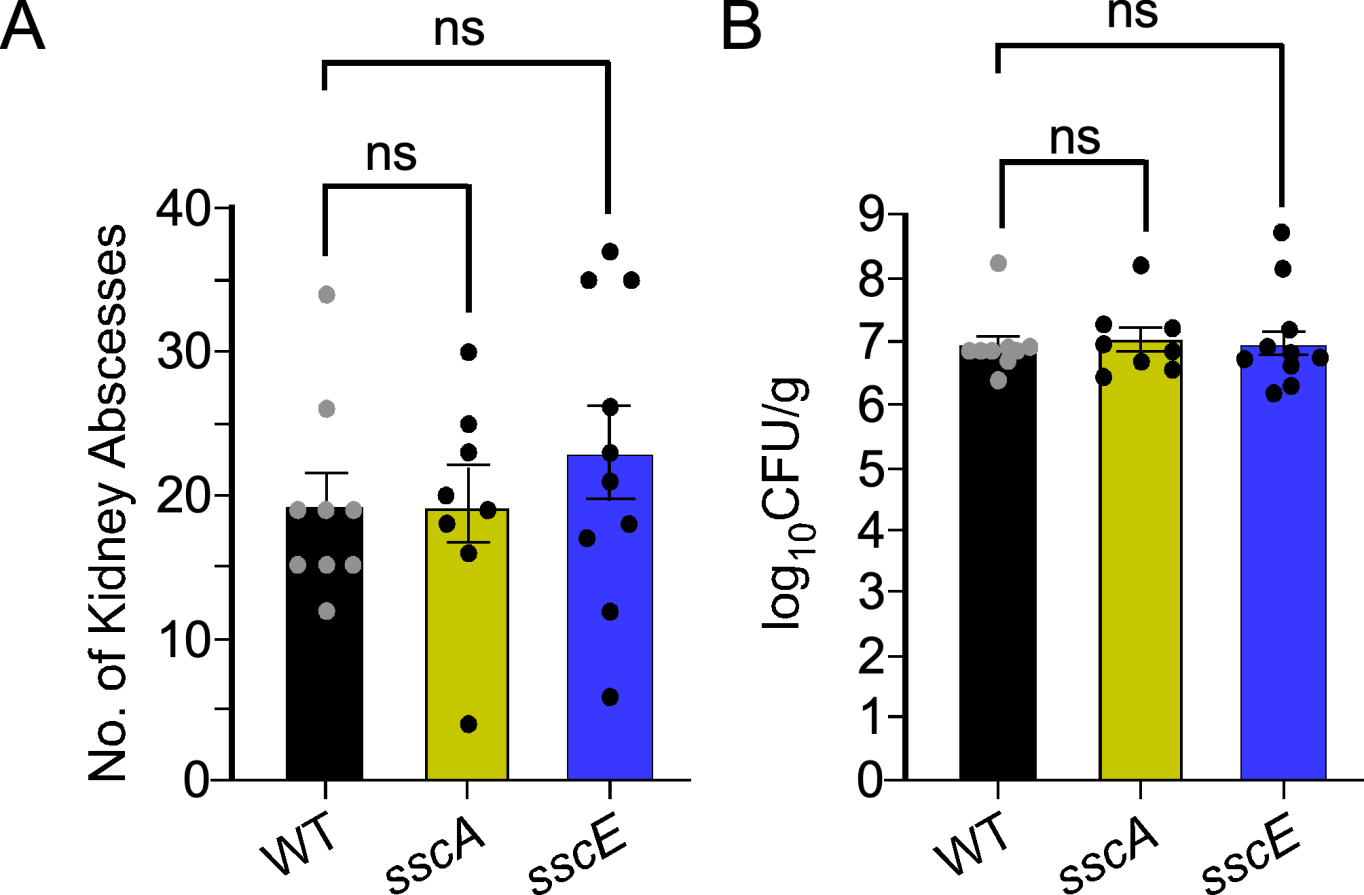
Genetic disruption of the Ssc biosynthesis pathway does not affect virulence in a bacteremia mouse model of infection. Mice (*n* = 8–10) were challenged intravenously with 10^7^CFU of *S. aureus* strain Newman (WT), *sscA,* or *sscE* and sacrificed 15 days later to enumerate abscess lesions on the surface of kidneys (**A**)and log_10_CFU/g (colony forming units per gram) of ground kidneys (B). Data were analyzed with one-way analysis of variance (Dunnett’s multiple comparison test) and reproduced once.

The ability to survive in the bloodstream and disseminate to organ tissues to form persistent abscesses are distinguishable features of *S. aureus* (53–55). These virulence properties can be modeled in the mouse following retro-orbital injection of bacteria (54). To investigate whether the *ssc* gene cluster contributes to virulence, mice were infected intravenously with strain Newman and the *sscA* and *sscE* mutants. Animals were sacrificed 15 days post-infection and organs were removed to enumerate surface abscesses and colony forming unit (CFUs) following serial dilutions of ground tissues (Fig. 5A and B). We did not observe a significant difference in the number of surface abscesses or CFUs in the organs of mice infected with mutant strains compared to wild type. Thus, we conclude that the Ssc polymer is not required for the bloodstream dissemination of *S. aureus* to organ tissues in strain Newman.

## DISCUSSION

In *S. aureus*, at least 13 surface proteins are synthesized as precursors with YSIRK/GXXS signal peptides and are secreted across septal membranes into the cross-wall compartment of dividing cells (19, 56). Such spatial secretion results in the abundant deposition and broad distribution of these proteins over the staphylococcal surface (57). In contrast, surface proteins bearing canonical signal peptides are deposited with low abundance into polar peptidoglycan (19). In *S. aureus*, four other precursors, the LytN murein hydrolase (58), the giant protein Ebh (59), and the glycerol-ester hydrolases, GehA and GehB (60), also carry YSIRK/GXXS signal peptides. While a rationale for the septal secretion of glycerol-ester hydrolases has not been revealed, Ebh and LytN play distinct roles during cell division. Mutants lacking *ebh* display increased cell size and altered envelope integrity (59), while septal secretion of LytN promotes the cleavage of cross-wall peptidoglycan for the separation of daughter cells (58).

Genetic studies established that YSIRK/GXXS precursors, like canonical precursors, require cytoplasmic SecA for secretion across the plasma membrane (22, 35). Furthermore, it was found that mutants that cannot synthesize Glc_2_-DAG, the lipid anchor of LTA, no longer restrict the secretion of SpA into the cross-wall compartment (24). Chemical mutagenesis also identified an isolate with spatial defect for the secretion of YSIRK/GXXS precursors (35). Unfortunately, this phenotypic defect could not be narrowed down to a specific mutation as the chemical mutagen introduced too many nucleotide polymorphisms, yet this experiment provides evidence for *trans*-acting factors of septal secretion (35). Here, we exploited our arranged transposon library to screen for mutants that can no longer restrict the secretion of SpA into the cross-walls of dividing cells. During this process, we came across a cluster of five genes predicted to encode an extracellular polysaccharide recently proposed to contain N-acetylgalactosamine and named staphylococcal surface carbohydrate (41). We generated gene deletions for *sscA*, *sscB,* and *sscE*, and confirmed that septal secretion of SpA is lost in strains lacking *sscB* and *sscE*, but not *sscA*, and demonstrated that this phenotype was rescued in *sscB* and *sscE* complemented strains.

Our NCBI BLAST search identified some similarities between gene products of the *scc* and *B. subtilis tua* clusters (Table 1). It has long been known that *B. subtilis* assembles multiple cell-bound anionic polymers (61). In *B. subtilis* strains 168 and W23, the most abundant polymer under normal growth condition is WTA. WTA consists of either poly(glycerol phosphate) [poly(GroP)] or poly(ribitol phosphate) [poly(RboP)] and is synthesized by the products of the *tag* genes (*tagABDEFGHO*) in strain 168 (62) and the *tar* genes (*tarABIJKLDF*) in strain W23 (63). In strain 168, two additional glycosyltransferases GgaA and GgaB polymerize poly(glucosyl N-acetylgalactosamine 1-phosphate) [poly(GlcGalNAc 1-P)] onto the linkage unit otherwise assembled by TagO, TagA, and TagB (64). Thus far, strains of *S. aureus* have been reported to assemble WTA consisting of either poly(GroP) or poly(RboP) but not poly(GlcGalNAc 1-P) (61, 65, 66). In *B. subtilis*, growth under phosphate-limited conditions results in the replacement of WTA with teichuronic acid, a phosphate-free carbohydrate polymer containing glucuronic acid (46, 67) that is synthesized by the enzymes encoded by the *tuaABCDEFGH* gene operon (45). In *B. subtilis* strain W23, teichuronic acid is composed of disaccharide repeating units of glucuronyl N-acetylgalactosamine, likely attached like WTA by a phosphodiester link to the C-6 of muramic acid (45). While we have not yet confirmed the composition of the Ssc polymer in *S. aureus*, we surmise that it is a phosphate-free carbohydrate similar to teichuronic acid. The function of teichuronic acid is not known but like WTA, this polymer could contribute to the bacterial cell shape and morphology (18, 68). In this study, we did not observe any replication defects when bacteria were grown in complex rich media. However, growth of the *sscA*, *sscB,* or *sscE* mutant was severely impacted in phosphate-limited condition. This finding indicates that the Ssc polymer could contribute key functions for viability under some growth conditions. Whether the Ssc polymer replaces WTA in phosphate-limited media remains to be determined. Despite this growth disadvantage, the mutants did not display a virulence defect in animals following bloodstream infection. Lei et al. found that MgrA down-regulates the expression of *ssc* genes (41). MgrA is a key transcriptional regulator of virulence (69); it is worth noting that MgrA also down-regulates the expression of several genes encoding cell wall-anchored proteins (including SpA) but upregulates the expression of genes encoding for capsule and secreted toxins (70).

**Fig 6:**
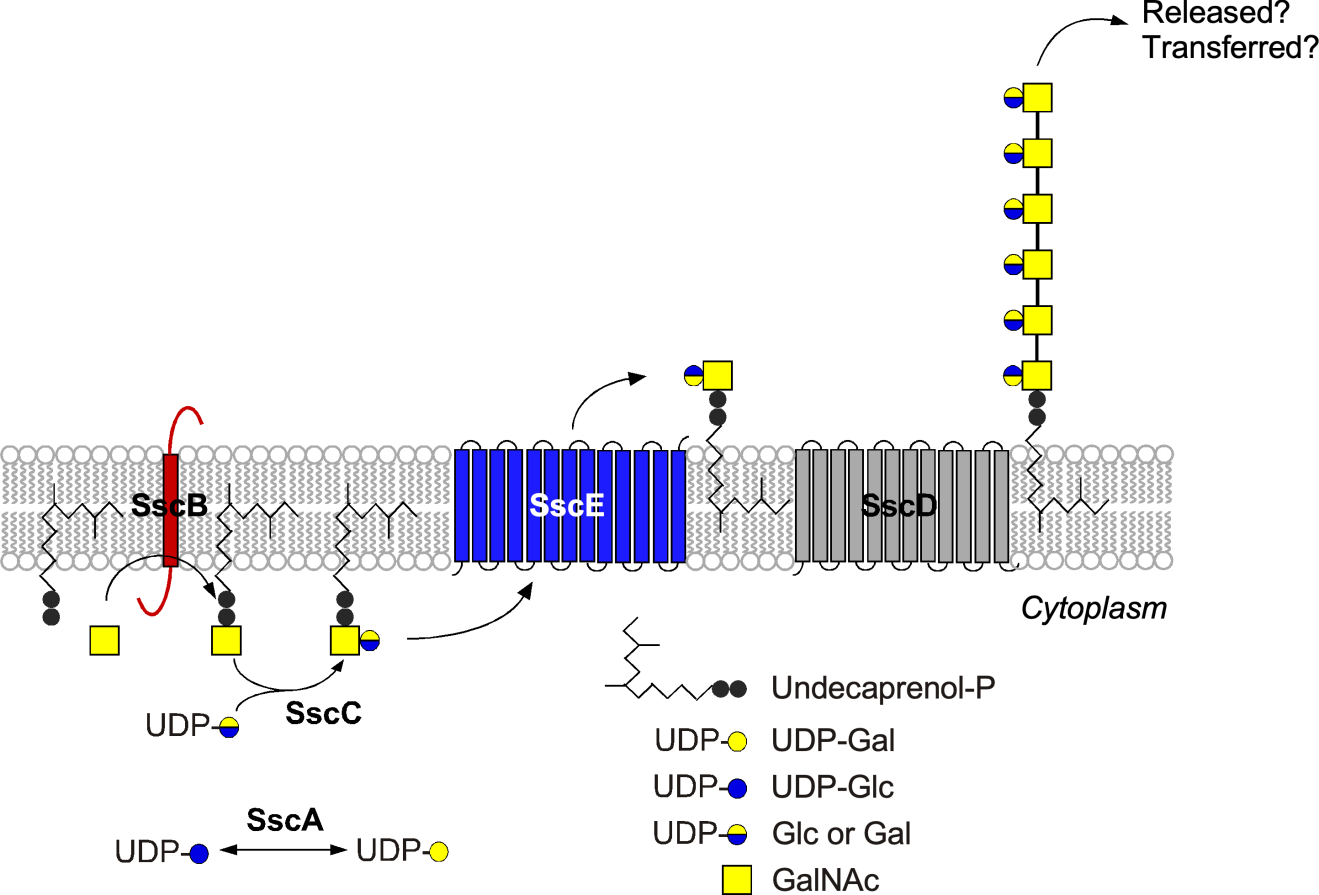
Proposed model for the assembly of the Ssc polymer in *S. aureus*.

Based on the extensive literature on assembly pathways of wall polymers and bioinformatic predictions, we can propose a model to account for the function of the *ssc* genes (Fig. 6). The predicted cytoplasmic domain of SscB shares homology with catalysts that transfer an activated sugar, possibly UDP-GalNAc, onto an undecaprenyl phosphate lipid carrier forming a lipid intermediate. We propose that this intermediate is modified with galactose or glucose by the predicted group 1 family glycosyltransferase SscC. Next, SscE flips the disaccharide bound to its lipid carrier to the *trans* side of the plasma membrane. Here, incoming subunits serve as substrates for the polymerization of disaccharide repeats by SscD, a predicted polymerase (Wzy_C homolog), onto undecaprenol-phosphate. In absence of cytoplasmic SscA, we surmise that unsubstituted poly(GalNAc) is still assembled and sufficient to support spatial secretion of precursors bearing a YSIRK/GXXS motif. However, in the absence of the glycopolymer or because of the accumulation of intermediates, spatial secretion of precursors bearing a YSIRK/GXXS motif is no longer restricted to septal membranes. Perhaps, not the polymer itself but the Ssc proteins interact with components of the cell division machinery along with factors that drive septal secretion. Future work will need to address these possibilities and establish whether the Ssc polymer may play a more critical role in the absence of WTA.

## MATERIALS AND METHODS

### Bacterial strains and growth conditions

Tryptic soy broth and agar were used to grow *S. aureus* strains. Lysogeny broth and agar were used to grow *E. coli* strains. When necessary, spectinomycin, ampicillin, and chloramphenicol were used at 200, 100, and 10 µg/mL, respectively. Modified M63 medium (71) was used to test phosphate limitation by altering the concentration of KH_2_PO_4_. Bacterial cultures were grown overnight in modified M63 medium containing 100 mM KH_2_PO_4_, then diluted (1:100) into fresh modified M63 medium containing either 100 mM or 0.25 mM KH_2_PO_4_. All growth curves were performed in triplicate in 96-well plates using a TECAN plate reader, and experiments were performed at least three times.

### Plasmids and strains

Plasmids, strains, and primers used in this study are listed in Table S1 and S2. Transposon mutants were from our Phoenix library collection (43) and arranged as per the order of genes on the chromosome of strain Newman (44). Newman and RN4220 mutant strains lacking genes *sscA* (NWMN0072/SAOUHSC_00088), *sscB* (NWMN0073/SAOUHSC_00089), and *sscE* (NWMN0076/SAOUHSC_00092), gene annotations are for Newman/RN4220 according to references (44, 72), were generated by allelic replacement using the pKOR1 vector (73). Briefly, 1 kb fragments upstream and downstream of the gene of interest were amplified by PCR using primer pairs listed in Table S2 to create a full deletion of the target gene by allelic replacement as described (73). In the case of *sscA*, the coding sequence of the gene was replaced with a spectinomycin resistance cassette. Complementation studies were conducted using the shuttle vector pSEW016 with the in-built Shine-Dalgarno and promoter sequences of the *S. aureus hprK* gene (74). The *sscB* and *sscE* genes were amplified from genomic DNA prepared from strain Newman and cloned into the *Sac*I and *BamH*I restriction sites of pSEW016. All mutants and complementing plasmids were confirmed by DNA sequencing.

### mRNA extraction and semi-quantitative PCR

A semi-quantitative PCR was used to check for the expression of *scc* genes. Briefly, RNA was extracted from strain Newman using RNeasy Mini Kit (Qiagen) following the manufacturer’s protocol. Next, RNA was reversed transcribed to cDNA using SuperScript IV Reverse Transcriptase kit (Invitrogen) and amplified using primers listed in Table S2.

### Microscopy

Microscopy experiments were performed as previously described (24, 75). For screening of the arranged Phoenix library, transposon mutants were grouped in batches of five along the chromosome of strain Newman. Briefly, cultures were grown in tryptic soy broth supplemented with the appropriate antibiotics when needed and collected when the absorbance of 600 nm (*A*_600_) reached a value between 0.5 and 0.6. Cells were pelleted by centrifugation for 3 min and washed once with phosphate-buffered saline, pH 7.4 (PBS) then subjected to sonication for 30 seconds to separate cells and avoid clustering. Cells were treated with trypsin 0.5 mg/mL (Sigma, USA) in 1 mL PBS suspension and incubated with rotation for 1 h at 37°C. Following trypsin treatment, cells were washed twice with PBS and suspended in tryptic soy broth supplemented with antibiotics when needed and 2.5 mg/mL soybean trypsin inhibitor (Sigma, USA) prior to incubation at 37°C with rotation. After 20 (*T*_20_) or 40 (*T*_40_) min, cells were collected by centrifugation and suspended in PBS. Two hundred fifty microliters of the cell suspension was transferred to a new tube for immediate fixing at room temperature for 20 min, with 250 µL of 2.5% paraformaldehyde and 0.006% glutaraldehyde in PBS. Cells were washed twice with PBS and were thoroughly vortexed to avoid cell clumping, before 150µL of the cell suspension was applied to eight-well poly-L-lysine-coated chamber coverslip (Ibidi, USA). After 10min of incubation, excess cells were removed by suction and wells were washed with PBS. Immobilized cells were blocked with 3% bovine serum albumin (BSA) in PBS for 1 h, followed by incubation with SpA-specific humanized monoclonal antibody (1:20,000 dilution in 3% BSA/PBS) overnight at 4°C. The next day, the cells were washed eight times with PBS and incubated with AF647-conjugated anti-human IgG (1:500 in 3% BSA/PBS) (Invitrogen, USA) for 3 h at room temperature in the dark. Samples were washed 10 times with PBS to remove excess unbound AF647 then incubated with 1 µg/mL BODIPY-FL vancomycin (Invitrogen, USA) for 10 min at room temperature in the dark followed by washing five times with PBS to remove excess dye. The slides were allowed to dry completely before ProLong Diamond Antifade Mountant (Invitrogen, USA) was added. Fluorescent images were immediately visualized and captured on a Leica Stellaris STED Confocal microscope with 63× oil objective. Identical settings and laser intensities were applied to all samples.

### Culture fractionation and immunoblotting

LTA extraction was performed as previously described (15). Briefly, overnight cultures were normalized to *A*_600_ of 3. One milliliter of cell suspensions was mixed with 0.2 g of glass beads, and bacteria were lysed by eight rounds of bead beating for 60 seconds each, with 5 min incubation periods on ice. Suspensions were centrifuged at 200 × *g* for 5 min to remove the glass beads, and 0.5 mL of the lysed cells transferred to new tubes. Bacterial membranes and LTA were sedimented by centrifugation at 16,000 × *g* for 15 min. Pellets were resuspended in 1× SDS sample buffer [62.5 mM Tris-HCl (pH 6.8), 2% SDS, 10% glycerol, 5% 2-mercaptoethanol, 0.01% bromophenol blue], boiled for 20 min at 95°C, separated on 15% SDS-PAGE gels, and transferred onto nitrocellulose membranes for western blotting. To analyze protein contents, whole cultures or washed cells obtained following centrifugation at 20,000 x *g* for 5 min were lysed with 20 µg/mL lysostaphin for 30 min at 37°C. Spent medium of cultures (supernatants) were transferred to fresh tubes. Proteins in all samples were precipitated using 10% trichloroacetic acid on ice for 1 h. Following centrifugation for 10 min at 20,000 × *g,* precipitates were washed with ice-cold acetone, dried, and solubilized in 100 µL 1× SDS sample buffer. Samples were boiled at 95°C for 10 min prior to SDS-PAGE separation followed by transfer onto nitrocellulose membrane. For immunoblotting experiments, all membranes were blocked using 5% milk in TBST [50 mM Tris-HCl (pH 7.5), 150 mM NaCl, 0.1% Tween 20] for 1 h and incubated overnight at 4°C with primary antibodies; mAb 55 (Novus Biologicals) was used to probe for LTA, in-house monoclonal antibodies were used to probe for SpA, and polyclonal sera were used to probe for LtaS, SasF, and SrtA. The blots were washed three times for 5 min with TBST before incubation with either secondary anti-mouse or anti-rabbit IgG linked to horseradish peroxidase (HRP) (Cell Signaling Technology, USA) to probe for LTA, LtaS, SasF, and srtA, or secondary anti-Human IgG (H + L) HRP Conjugate (Promega, USA) to probe for SpA. After 1 h incubation, the blots were washed three times with TBST, developed using SuperSignal West Pico Plus Chemiluminescence Substrate (ECL solution) (Thermo Scientific, USA), and visualized using Fotodyne imaging system.

### Antibiotic susceptibility testing

MICs of antibiotics were tested according to the Clinical and Laboratory Standards Institute (CLSI) guidelines using either broth microplate assay or MIC strip test (76). Briefly, inocula were adjusted to 5 × 10^5^ CFU/mL and added to 96-well plates supplemented with serially diluted antibiotics at a starting concentration of 256 to 0.125 µg/mL. The experiment was repeated at least twice, and MICs were recorded both visually and using the GENios plate reader. For MIC strip test, the inoculum was spread on the surface of Mueller-Hinton agar plates, then MIC strips purchased from Liofilchem were placed in the middle of the plate followed by incubation at 37°C for 18 h.

### Mouse renal abscess model

BALB/c mice were obtained from Jackson Laboratory at 6 weeks of age and divided in cohorts of 10 for sub-lethal challenges with *S. aureus* strains. Cultures were grown to *A*_600_ of 0.41, bacteria were washed once and re-suspended in sterile PBS. Animals were anesthetized by isoflurane inhalation and injected into the periorbital venous plexus with 100 µL of the bacterial suspension corresponding to an inoculum of 1 × 10^7^ CFU. Mice were monitored for clinical signs of disease, and body weight was recorded daily for up to 15 days. On days 5 and 15, five mice from each group were euthanized by carbon dioxide inhalation, both kidneys were collected for either CFU or surface abscess enumerations. The staphylococcal load in kidneys was analyzed by homogenizing renal tissues with PBS containing 0.1% Triton X-100 and plating serial dilutions of homogenate on tryptic soy agar (TSA) plates.

### Statistical analysis

Two-way analysis of variance (ANOVA) using Dunnett’s multiple comparison test was used to analyze data for the growth defect at 20 h in phosphate-limited conditions. For microscopy analysis, cells from three different fields of three different biological experiments were counted using ImageJ software (77). Septal versus aberrant Spa signal was calculated as a percentage of total cells counted. Data were analyzed using GraphPad Prism software, and two-way ANOVA with Dunnett’s multiple comparison test was used to compare the mean (SEM) for each mutant or complemented strain with the mean of wild-type cells. For immunoblots, data were generated from at least three biological repeats, and one-way ANOVA with Dunnett’s multiple comparison test was used to analyze the data. The relative abundance of proteins or LTA was determined by densitometry of blots using signals of samples from wild-type extracts as 100%. Housekeeping protein SrtA in wild-type samples was used for normalization, and immune signals for the mutants were divided by that of wild type then multiplied by 100. Percentage of released SpA was measured by dividing values for released SpA over total signals (cell associated + released) then multiplying by 100. Lastly, one-way ANOVA using Dunnett’s multiple comparison test was used to analyze data for the mouse infection study.

## SUPPLEMENTAL MATERIAL

Supplemental figures and tables jb.00478-24-s0001.docx Fig. S1 to S3; Tables S1 and S2.

ASM does not own the copyrights to Supplemental Material that may be linked to, or accessed through, an article. The authors have granted ASM a non-exclusive, world-wide license to publish the Supplemental Material files. Please contact the corresponding author directly for reuse.
