# Supplementary material for: A novel polysaccharide in the envelope of S. aureus influences the septal secretion of preproteins with a YSIRK/GXXS motif
Source: J Bacteriol. 2025 Jan 28;207(2):e00478-24. doi: 10.1128/jb.00478-24 (PMC11841062; doi:10.1128/jb.00478-24)
Supplement: Supplemental figures and tables — Fig. S1 to S3; Tables S1 and S2. [file jb.00478-24-s0001.docx]

**Supporting Information for**

**A novel polysaccharide in the envelope of *S. aureus* influences the septal secretion of preproteins with a YSIRK/GXXS motif**

Amany M. Ibrahim and Dominique Missiakas^#^

Department of Microbiology, Howard Taylor Ricketts Laboratory, The University of Chicago, Lemont, Illinois, USA

#Address correspondence to Dominique Missiakas, [dmissiak@bsd.uchicago.edu](mailto:dmissiak@bsd.uchicago.edu)

**This file contains:**

- Supplementary Figures 1-3
- Supplementary Tables 1-2


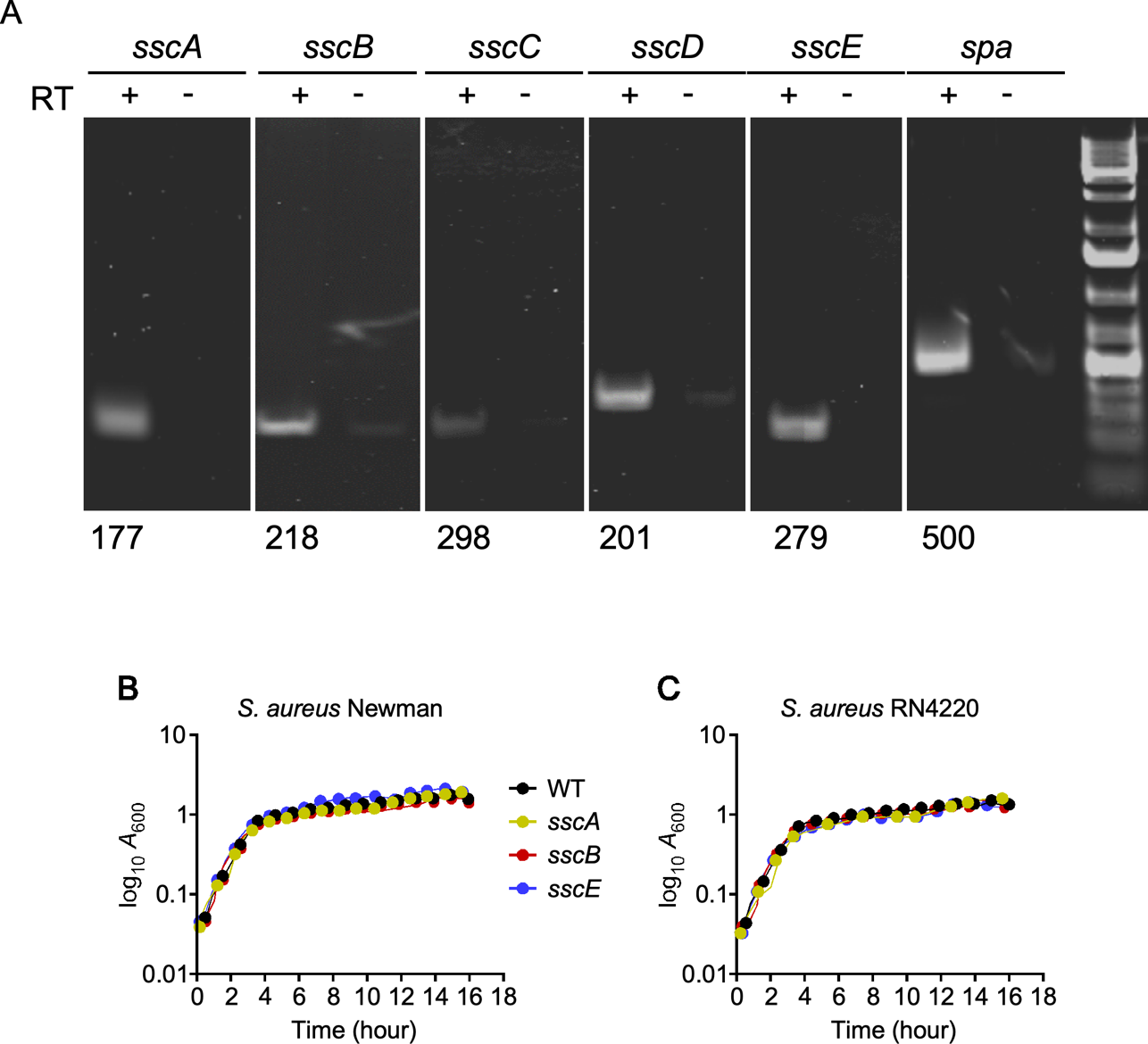


**Fig. S1.** Characterization of the *ssc* genes. (A) Analysis of *ssc* gene expression. Wild type Newman cells were grown to mid log phase (*A*_600_=0.6) then subjected to mRNA extraction using RNeasy Mini Kit (Qiagen). mRNA was reverse transcribed into cDNA then a semi-quantitative PCR was conducted with primers specific to each gene product. The expression of the *spa* gene was used as a positive control, while RNA samples without addition of reverse transcriptase (-RT) were used as a negative control. Numbers under the gels indicate the size of amplified DNA fragment in base pairs. (B, C) Growth curves of wild type (Newman, B; RN4220, C) and isogenic *sscA*, *sscB*, and *sscE* mutants. Growth was monitored as change in absorbance at 600 nm (*A*_600_).

**
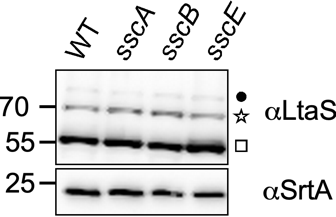
**

**Fig. S2. LtaS processing is not impaired in the *ssc* mutants.** LtaS processing was examined by immunoblotting samples from washed cells as reported in Fig. 4A. Blots were analyzed with antibodies against LtaS (αLtaS) and sortase A (αSrtA) as a loading control. Experiments were performed at least three times. The star and square identify the LtaS precursor (MW 70 kDa) and mature protein (MW 55 kDa), respectively, while the dot identifies an unknown protein cross-reactive with αLtaS. The sizes of the MW markers are shown to the left of each blot.


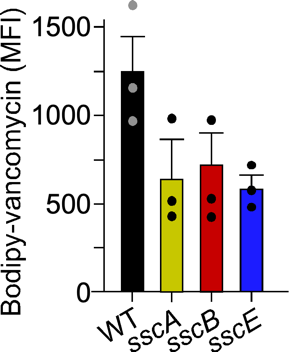


**Fig. S3. Quantification of BODIPY-vancomycin fluorescence from micrographs as shown in Fig. 2A.** The mean fluorescence intensity (MFI) of the vancomycin (green) channel from three different fields of two biological repeats was quantified using ImageJ after subtracting the background signal. Data were analyzed using one-way ANOVA with Dunnett's multiple comparisons test. No significant differences were observed despite a mild reduction in the vancomycin signal for the *ssc* mutants.

**Table S1.** Bacterial strains or plasmids used in this study.

| **Name and designation** | **Description** | **Source or reference** |
| --- | --- | --- |
| ***Vectors and plasmids*** | | |
| pSEW016 | *E. coli/S. aureus* shuttle vector | Laboratory collection |
| pKOR1 | Allelic replacement vector | (1) |
| pKOR1-*SAOUHSC_00088:spec* | Δ*SAOUHSC_00088:spec* gene deletion cloned into pKOR1 | This study |
| pKOR1-*SAOUHSC_00089* | Δ*SAOUHSC_00089* gene deletion cloned into pKOR1 | This study |
| pKOR1-*SAOUHSC_00092* | Δ*SAOUHSC_00092* gene deletion cloned into pKOR1 | This study |
| p*sscA* | Gene NWMN_0072 cloned into pSEW016 | This study |
| p*sscB* | Gene NWMN_0073 cloned into pSEW016 | This study |
| p*sscE* | Gene NWMN_0076 cloned into pSEW016 | This study |
| **Strains** | | |
| DH5α | *E. coli* cloning strain | Laboratory collection |
| RN4220 | *S. aureus* wild type (WT) strain | Laboratory collection |
| Newman | *S. aureus* wild type (WT) strain | Laboratory collection |
| ΔSAOUHSC_00088 (*sscA*) | RN4220 with SAOUHSC_00088 gene deletion | This study |
| ΔSAOUHSC_00089 (*sscB*) | RN4220 with SAOUHSC_00089 gene deletion | This study |
| ΔSAOUHSC_00092 (*sscE*) | RN4220 with SAOUHSC_00092 gene deletion | This study |
| ΔNWMN_0072 (*sscA*) | Newman with NWMN_0072 gene deletion | This study |
| ΔNWMN_0073 (*sscB*) | Newman with NWMN_0073 gene deletion | This study |
| ΔNWMN_0076 (*sscE*) | Newman with NWMN_0076 gene deletion | This study |
| *sscA*/p*sscA* | ΔNWMN_0072 complemented with p*sscA* | This study |
| *sscB*/p*sscB* | ΔNWMN_0073 complemented with p*sscB* | This study |
| *sscE*/p*sscE* | ΔNWMN_0076 complemented with p*sscE* | This study |

**Table S2.** Oligonucleotide sequence of primers used in this study.

| **Primer name** | **Purpose** | **Sequence** |
| --- | --- | --- |
| Spec0072-F | Replacement of *NWMN_0072* with spec cassette | GCGCTCGAGATCGATTTTCGTTCGTGAATAC |
| Spec0072-R |  | GCGCCATGGGATATGCAAGGGTTTATTGTTTTCTAAAATC |
| pK0072F1-F | Knocking out *NWMN_0072* | GGGGACAAGTTTGTACAAAAAAGCAGGCTGCAGCTGCACTTAAATTATCAAGTG |
| 0072F1-R |  | GCGCTCGAGCTCATTTATACATTAAAAATATATCATAAAAACATAAAGTATTGTAAG |
| 0072F2-F |  | GCGCCATGGAAAATGACATTGAAGCTGTCCATAATAATAAG |
| pK0072F2-R |  | GGGGACCACTTTGTACAAGAAAGCTGGGTGTTGTTCAATCATTGGTCCATGATTG |
| pK0073F1-F | Knocking out *NWMN_0073* | GGGGACAAGTTTGTACAAAAAAGCAGGCTATGTTTTTATGATATATTTTTAATGTATAAATGAGGTG |
| 0073F1-R |  | GCGCTCGAGTATTATCTACCTCAAAATTAAAGTAATCCTTTAAAC |
| 0073F2-F |  | GCGCTCGAGCTTAATTTACTTAATAAGTTCAAATAAAAGTTATATTTTAAAG |
| pK0073F2-R |  | GGGGACCACTTTGTACAAGAAAGCTGGGTCTTGTTGCTGACAATCATTTAATGTAGGTC |
| pK0076F1-F | Knocking out *NWMN_0076* | GGGGACAAGTTTGTACAAAAAAGCAGGCTTTGCTATTTTTTGATGATTTGATATTTATTACGGTTAAGG |
| 0076F1-R |  | GCGCTCGAGTACGATCCCTTTCCATCTTTTCATATTGAAC |
| 0076F2-F |  | GCGCTCGAGGTACCGGTAATGCTATACTTTAGAAAATTAAGATTAAG |
| pK0076F2-R |  | GGGGACCACTTTGTACAAGAAAGCTGGGTGAGTTGAAATTTATCTTATTATTTTTCCACTTTTACGTG |
| WT0073-F | Δ*NWMN_0073* complementing vector | GCGCGAGCTCATTGAAGAAGTTACAGCTAAAGAAGTGG |
| WT0073-R |  | GCGCGGATCCCTACCTTGAGCCTTCCCCTG |
| WT0076Comp-F | Δ*NWMN_0076* complementing vector | GCGGAGCTCATGAAGAGTGATTCACTAAAAGAAAATATTATTTATCAAGGG |
| WT0076Comp-R |  | GCGGGATCCTTAAATTGTTTTATGTCGAAGATGCCTCAATATTTG |
| RNA0072-F | Detecting *NWMN_0072* expression | TGGCTGACGATCATGTGTTTG |
| RNA0072-R |  | ACAATCTTAATGTTGCTACGACG |
| RNA0073-F | Detecting *NWMN_0073* expression | TGAAGCTGTCCATAATAATAAGGGT |
| RNA0073-R |  | CGCACCGTTTTTCTCTGCG |
| RNA0074-F | Detecting *NWMN_0074* expression | CGATTGAAATTAACCACAATACATAATGGTATTG |
| RNA0074-R |  | CTTGTTGCTGACAATCATTTAATGTAGG |
| RNA0075-F | Detecting *NWMN_0075* expression | GTATACATATCGTATGATTATTACGCTTTGTTTGC |
| RNA0075-R |  | CCTATAGTCACACTGCTTATAATATAAGAGGTAACG |
| RNA0076-F | Detecting *NWMN_0076* expression | GCGTTAACAGTTTTTGCGATGTATATGG |
| RNA0076-R |  | CAAAGGTAATTGGTTTAATACCGTCACAATAGC |

**SI References**

1. Bae T & Schneewind O (2006) Allelic replacement in Staphylococcus aureus with inducible counter-selection. *Plasmid* 55(1):58-63.
